# Effect of Social Vulnerability Index on Betamethasone Timing in Patients at Risk of Preterm Birth

**DOI:** 10.3390/jcm13247798

**Published:** 2024-12-20

**Authors:** Lizelle Comfort, Gillian Piltch, David Krantz, Frank Jackson, Matthew J. Blitz, Burton Rochelson

**Affiliations:** 1 Northwell, New Hyde Park, NY 11040, USA; gpiltch@northwell.edu (G.P.); dkrantz@northwell.edu (D.K.); mblitz@northwell.edu (M.J.B.); brochels@northwell.edu (B.R.); 2 Department of Obstetrics and Gynecology, North Shore University Hospital, Manhasset, NY 11030, USA; 3 Department of Obstetrics and Gynecology, Long Island Jewish Medical Center, New Hyde Park, NY 11030, USA; 4 Zucker School of Medicine at Hofstra/Northwell, Hempstead, NY 11549, USA; 5 Northwell Health Laboratories, Lake Success, NY 11042, USA; 6 Department of Obstetrics and Gynecology, South Shore University Hospital, Bay Shore, NY 11706, USA

**Keywords:** preterm birth, antenatal corticosteroids, betamethasone, social vulnerability index

## Abstract

**Background/Objectives:** Several social vulnerability index (SVI) components have been associated with adverse obstetrical outcomes and provider bias. The objective of this study is to assess whether betamethasone administration timing among patients at risk for preterm birth differs by social vulnerability index. **Methods:** A multicenter retrospective cohort study of pregnant people at a large academic healthcare system between January 2019 and January 2023. Patients with live singleton gestations at risk for preterm birth who received at least one dose of intramuscular betamethasone for fetal lung maturity from 22 to 34 weeks were included. Patients aged less than 18, who received late-preterm corticosteroids and/or had scheduled delivery at 34 weeks were excluded. We analyzed the association between patient SVI quartile and maternal demographic factors on betamethasone timing, with optimal timing defined as the receipt of two doses of betamethasone within 2 to 7 days of delivery. **Results:** 1686 patients met the inclusion criteria. Only 22.4% of patients had optimally timed betamethasone administration. Among those who did not receive optimal betamethasone timing, 360 patients delivered less than 48 h from the first dose and 948 delivered greater than 7 days from the first dose. Optimal betamethasone timing within 2 to 7 days of delivery was more common in patients with higher SVI values. Patients with lower social vulnerability were more likely to deliver greater than one week from betamethasone administration. **Conclusions:** Patients in higher SVI quartiles are more likely to have optimally timed betamethasone. This is likely attributed to overtreatment with betamethasone of less socially vulnerable populations.

## 1. Introduction

Preterm birth, defined as delivery between 20 weeks gestation and 36 weeks 6 days gestation, is an obstetrical complication associated with long-term implications for perinatal and childhood health [1]. Interventions in the case of a high likelihood of preterm birth include the administration of antenatal corticosteroids for fetal lung maturity [1], which is associated with significant reduction in neonatal morbidity and mortality when used appropriately. Intramuscular betamethasone is the most commonly utilized antenatal corticosteroid in the United States and is administered in two 12-milligram doses given 24 h apart in preterm patients with a high likelihood of imminent delivery [2]. A significant body of evidence supports the administration of antenatal corticosteroids in women at risk of preterm birth prior to 34 weeks of gestation to reduce complications of prematurity including respiratory distress syndrome, necrotizing enterocolitis, and intraventricular hemorrhage [3]. In particular, the benefits in reducing respiratory distress syndrome are greatest in preterm infants born at 28 to 34 weeks gestation. Outcomes are optimized when maternal antenatal corticosteroid administration occurs within 7 days of preterm birth [4]. In practice, the administration of antenatal corticosteroids is frequently sub-optimally timed, particularly among patients at risk of spontaneous preterm birth [5,6]. Providers may have a low threshold to administer antenatal corticosteroids given that improved neonatal outcomes are seen when antenatal corticosteroids are administered at least 2 days prior to delivery [7].

Preterm birth, like many obstetrical complications, is associated with racial, ethnic and socioeconomic disparities [1]. Social vulnerability is a term that refers to demographic and socioeconomic factors that may adversely affect communities [8]. Several of these components have been independently associated with a variety of adverse obstetrical outcomes [9], including an increased risk of preterm birth. The four domains that comprise the social vulnerability index (SVI) are socioeconomic status, household composition and disability, minority status and language, and housing and transportation [10]. Social vulnerability arises out of differential social relations among groups within a society [11], including geographic localization, race/ethnicity and other patient-level factors such as cultural origin, immigration, exposure to interpersonal violence, psychological distress, linguistic barriers and addictions [12].

Living in an area with a higher overall SVI score and higher social vulnerability in each individual SVI theme has been associated with an increased risk of preterm birth [13]. However, another study in a heterogenous cohort did not demonstrate a statistically significant difference in a composite of adverse pregnancy outcomes based on SVI score [14]. Social vulnerability may also be associated with inherent bias in patient–provider interactions [15,16] and individual variability in practices among obstetrician gynecologists [17].

Despite associations between SVI values and rates of preterm birth, few studies have explored the association between patient SVI score and provider variability in the management of preterm birth. One retrospective study found no significant differences between patients who received antenatal corticosteroids within 7 days and greater than 7 days regarding baseline maternal characteristics including race/ethnicity [18]. However, this was a small retrospective study, and social vulnerability was not examined directly. Other studies have found that Black mothers are significantly less likely to receive antenatal corticosteroids than White mothers [19,20].

Given the association of social vulnerability and the risk of preterm birth, implicit biases that may impact the patient–provider relationship and care management, and emerging data on social vulnerability overall, the objective of this study was to assess the impact of patient SVI scores on the timing of antenatal corticosteroid administration among patients at risk for preterm birth. We hypothesized that the successful optimal timing of antenatal corticosteroids for spontaneous preterm birth would differ by social vulnerability factors.

## 2. Materials and Methods

We performed a multicenter retrospective cohort study of pregnant patients who received betamethasone for fetal lung maturity from 22 to 34 weeks gestation at seven birthing hospitals in a large academic healthcare system between January 2019 and December 2022. Pregnant patients with singleton gestation who were at risk for preterm birth (for example, in the setting of preterm prelabor rupture of membranes, preterm labor, pre-eclampsia with severe features, or other medical indication for iatrogenic preterm delivery) and who received at least one dose of intramuscular betamethasone for fetal lung maturity were included. Patients who were less than 18 years old or had medical contraindications to steroid administration (active diabetic ketoacidosis, need for emergent delivery), multifetal gestations, fetal demise, or lethal fetal anomalies were excluded. We also excluded patients who received late-preterm steroids (betamethasone course administered beginning on or after 34 weeks gestation) or who had a scheduled delivery at 34 weeks gestation and received betamethasone in the week prior to delivery, as these patients by default had optimal timing of antenatal corticosteroid administration.

The following maternal demographic data were collected from the electronic medical record: delivering hospital location, maternal age at delivery, gravidity and parity, number of previous preterm deliveries (defined as history of delivery between 20 weeks gestation and 36 weeks 6 days gestation), insurance status (receipt of Medicaid or private insurance), maternal body mass index (BMI, kg/m^2^), primary language, marital status, patient-identified race/ethnicity, obstetrical comorbidity index score [21], hypertensive disorder of pregnancy, maternal substance use and delivery information. Race and ethnicity were chosen for comparison given known disparities in preterm birth rates among different racial or ethnic subgroups.

The patient’s address of residence was used to identify their census tract and then linked to SVI scores released by the Center for Disease Control and Agency for Toxic Substances and Disease Registry [8]. No patients were missing SVI data. The SVI score incorporates 16 U.S. census variables that fall into four major themes: socioeconomic status (income below 150% poverty level, unemployment, education, health insurance), household characteristics (children, senior, disability, single-parent, English language proficiency), racial and ethnic minority status, and housing type and transportation (multi-unit structures, mobile homes, crowding, group quarters, no vehicle). Each census tract receives an overall score, as well as a score for each of the 4 themes, that is between 0 and 1. The higher the SVI score, the higher the social vulnerability of a community; for example, a ranking of 0.85 indicates that 85% of regions are less vulnerable than the region of interest and that 15% of regions are more vulnerable [8]. We report SVI scores in quartiles (quartile 1 = low vulnerability, quartile 2 = moderately low vulnerability, quartile 3 = moderately high vulnerability, quartile 4 = high vulnerability).

The dates and times of betamethasone administration were recorded. The following delivery information was collected: gestational age at delivery and whether the delivery was spontaneous (following admission for contractions, rupture of membranes, cervical dilation or vaginal bleeding) or medically indicated. The time difference between the first dose of betamethasone and delivery was calculated and recorded in days. Optimal timing of antenatal corticosteroids was defined as the receipt of 2 doses of betamethasone within 2 to 7 days of delivery, as per the American College of Obstetricians and Gynecologists guidelines [2]. A secondary analysis was performed for those who delivered between 6 h and 7 days following initial betamethasone administration, given that neonatal benefit may also occur with shorter intervals of antenatal corticosteroid administration [4,22].

Following access from the electronic medical record, the data were stored in a password-protected database. A de-identified, non-coded version of the database was used for statistical analysis. Categorical variables were tested for significance using the chi-square or Fisher’s exact test. Logistic regression was performed to identify whether optimal timing of betamethasone was associated with SVI score and individual patient factors. In addition, nominal logistic regression was performed to evaluate the less than 2 day and greater than 7 day groups with the optimal timing group. All odds ratios were converted so that the compared odds represented the ratio of the number in the optimal group to the associated number in the suboptimal group. Therefore, higher odds ratios indicate a greater likelihood of having had optimal timing. All statistical analyses were performed using SAS version 9.4M6 (Cary, NC, USA).

This study received institutional review board approval prior to data collection.

## 3. Results

A total of 1686 patients met the inclusion criteria. The following patients were excluded: 8 patients aged less than 18 years, 1226 patients who received betamethasone in the late preterm period (beginning 34 weeks gestation or later), 121 patients with a planned or scheduled delivery at 34 weeks gestation, 7 patients with fetal demise, and 2 patients who were less than 22 weeks gestation at delivery. There were multiple demographic differences based on SVI score: patients with higher social vulnerability index values were more likely to be non-White, have a higher BMI, have Medicaid insurance, be unmarried, speak a non-English language, and have a history of preterm delivery (Table 1). There were no significant differences in maternal age, gravidity/parity, reported substance use, or gestational age of betamethasone administration among the groups.

Only 22.4% of all patients received optimally timed betamethasone administration. Among those that did not receive optimal betamethasone timing, 360 patients delivered less than 48 h from the first dose and 948 delivered greater than 7 days from the first dose. Optimal betamethasone timing was more common in patients with higher SVI scores, with significantly more patients in the third and fourth SVI quartiles receiving optimal betamethasone timing (odds ratio 1.81 (1.25, 2.60)). This trend remained significant after controlling for the above maternal demographics, including age, gravidity/parity, BMI, insurance status, marital status, race/ethnicity, prior preterm birth, preferred language and history of substance use (adjusted odds ratio 1.49 (1.01, 2.22), Table 2). A time of betamethasone administration to delivery of 6 h to 7 days was also more common among patients with higher SVI scores (adjusted odds ratio for the highest SVI quartile 1.53 (1.09, 2.14)).

**Figure 1:**
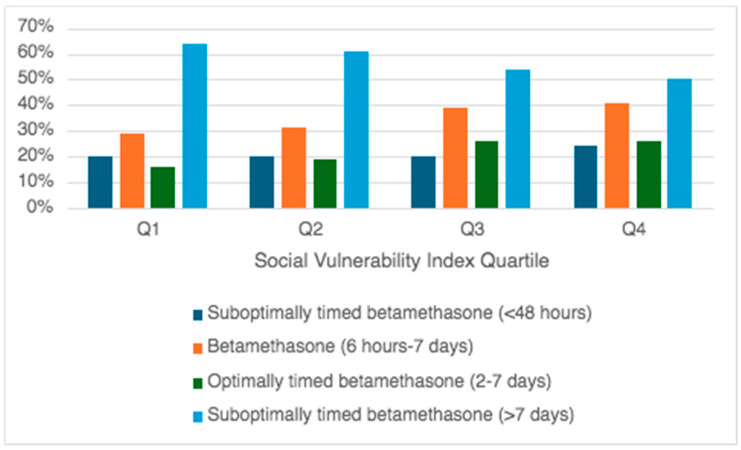
Timing of betamethasone administration by social vulnerability index quartile.

In examining more precise trends of betamethasone administration among patients who had suboptimal timing from administration to delivery, the rate of administration less than 48 h prior to delivery and less than 6 h prior to delivery did not differ by SVI quartile. However, patients of higher SVI quartiles (patients with increased social vulnerability) were less likely to receive betamethasone greater than seven days prior to delivery (Table 2). These trends were not gestational-age dependent. Figure 1 demonstrates the summary of betamethasone timing by different SVI quartiles.

We further evaluated the timing of betamethasone based on individual maternal demographics. Patients with higher parity (greater than one) were less likely to receive the optimal timing of betamethasone compared to nulliparous patients or patients with one prior delivery, whereas there was no difference in timing based on maternal age, BMI, marital status, language, insurance status, or maternal substance use (Supplemental Table S1). There did appear to be some differences based on race/ethnicity, with non-Hispanic Black patients and those who identify as “Other” race or ethnicity having higher unadjusted rates of optimal betamethasone administration; however, when adjusted for the baseline demographic variables found in Table 1 and social vulnerability index score, the differences in betamethasone timing did not remain significant among groups of different race/ethnicities (adjusted odds ratio 1.28 (0.91, 1.79)).

## 4. Discussion

In this study, patients with higher SVI values were more likely to receive optimally timed antenatal corticosteroids and were less likely to deliver greater than 7 days from the time of betamethasone administration. To our knowledge, this is the first study to examine the impact of patient social vulnerability on antenatal corticosteroid timing.

Our findings were somewhat unanticipated, and may reflect either that patients of higher SVI quartiles are a higher risk group for preterm birth, or that patients of lower social vulnerability are overtreated with betamethasone when they are not truly at a high risk of preterm birth. Additionally, within this study’s healthcare system, patients of higher SVI quartiles who receive government-funded insurance (Medicaid) are often cared for by medical trainees who may be more likely to adhere to institutional guidelines for antenatal corticosteroids compared to private practitioners or community physicians.

While antenatal corticosteroids are critical to reducing the complications of preterm neonates, the appropriate timing of betamethasone has been a longtime challenge for obstetrical providers. In clinical practice, steroids are typically administered to women at risk of delivery within 7 days, with the decision individualized based on the likelihood of preterm birth and the risks to mother and fetus. However, in the existing literature, most at-risk patients do not receive appropriately timed antenatal corticosteroids, with optimal timing occurring in about 20–40% of cases [5,6,18]. There have been differences in the management of preterm birth among at-risk patients based on maternal race, with previous studies demonstrating that Black pregnant patients are less likely to receive antenatal corticosteroids [19,20]. In this study, we did not find a difference in the rate of optimal timing of betamethasone based on race/ethnicity.

We did not evaluate neonatal outcomes in this study. Since betamethasone is initiated prior to 34 weeks gestation, a later gestational age at delivery is inherently associated with suboptimal betamethasone timing but improved neonatal outcomes. However, the existing literature does suggest the neonatal benefit of optimal antenatal corticosteroid timing, with neonates exposed to antenatal corticosteroids 2 to 7 days before delivery less likely to develop respiratory distress syndrome compared to neonates who received betamethasone less than 48 h prior to delivery or greater than or equal to 14 days prior to delivery [23]. Shorter intervals of administration to delivery may also be associated with neonatal benefit [22]. The risks of administration of antenatal corticosteroids greater than 7 days prior to delivery are not well described, with concerns of reduced neonatal birthweight and reduction in clinical benefit reported in the existing obstetrical literature [24]. The additional implications of unnecessary betamethasone administration include patient anxiety and the utilization of hospital system resources.

The strengths of this study include access to a heterogeneous population including a large number of patients with available SVI variables and demographic data. However, this study was limited by the retrospective study design, with optimal timing related only to the documentation of betamethasone administration in relationship to the date/time of delivery. This study also did not examine the timing of late-preterm steroids and only evaluated timing based on the initial administered dose of betamethasone; therefore, some patients in our cohort with inappropriate timing of the initial betamethasone course may have later had a second course that was appropriately timed. Additionally, we recognize that the social vulnerability index may be an imperfect measure that does not fully explain individual patient risk and/or vulnerability [25].

## 5. Conclusions

Individual social vulnerability factors have been independently associated with an increased risk of preterm birth and may influence patient–provider interactions. In this study, we found a difference in the practice of betamethasone administration for patients at risk of preterm delivery, with patients of higher social vulnerability less likely to receive a suboptimal timing of betamethasone; this was predominantly driven by the decrease in administration greater than 7 days to the time of delivery.

We continue to advocate for the judicious administration of antenatal corticosteroids to patients truly at increased risk of imminent preterm birth. The identification of the impact of social vulnerability on preterm birth management may increase provider awareness and potentially mitigate social barriers to obstetrical care. Future directions include the further exploration of patient–provider interactions that may influence differences in care in order to identify and eliminate bias in clinical practice.

## Figures and Tables

**Table 1:** Patient baseline demographic data.

Characteristic	All (N = 1686)	Q1 (N = 298)	Q2 (N = 407)	Q3 (N = 445)	Q4 (N = 536)	*p* *
Maternal age						
<35	1063 (63.1%)	172 (57.7%)	244 (60.0%)	290 (65.2%)	357 (66.6%)	0.12
35–39	438 (26.0%)	85 (28.5%)	113 (27.8%)	112 (25.2%)	128 (23.9%)	
>=40	185 (11.0%)	41 (13.8%)	50 (12.3%)	43 (9.7%)	51 (9.5%)	
BMI						
18–29	747 (44.8%) [n = 1668]	157 (53.2%) [n = 295]	171 (43.0%) [n = 397]	202 (45.6%) [n = 443]	217 (40.7%) [n = 533]	0.02
30–39	756 (45.3%) [n = 1668]	116 (39.3%) [n = 295]	186 (46.9%) [n = 397]	207 (46.7%) [n = 443]	247 (46.3%) [n = 533]	
>=40	165 (9.9%) [n = 1668]	22 (7.5%) [n = 295]	40 (10.1%) [n = 397]	34 (7.7%) [n = 443]	69 (13.0%) [n = 533]	
Gestational age at initial betamethasone dose						
<28 weeks	408 (24.2%)	58 (19.5%)	95 (23.3%)	111 (24.9%)	144 (26.9%)	0.28
28–32 weeks	689 (40.9%)	134 (45.0%)	171 (42.0%)	171 (38.4%)	213 (39.7%)	
32–33 weeks	589 (34.9%)	106 (35.6%)	141 (34.6%)	163 (36.6%)	179 (33.4%)	
Race/ethnicity						
Non-Hispanic White	496 (29.4%)	167 (56.0%)	150 (36.9%)	94 (21.1%)	85 (15.9%)	<0.01
Non-Hispanic Black	469 (27.8%)	36 (12.1%)	102 (25.1%)	133 (29.9%)	198 (36.9%)	
Asian or Pacific Islander	300 (17.8%)	38 (12.8%)	58 (14.3%)	104 (23.4%)	100 (18.7%)	
Other/unknown	421 (25.0%)	57 (19.1%)	97 (23.8%)	114 (25.6%)	153 (28.5%)	
Gravidity						
1	470 (27.9%)	86 (28.9%)	114 (28.0%)	129 (29.0%)	141 (26.4%)	0.01
2–3	786 (46.6%)	143 (48.0%)	145 (51.6%)	198 (44.5%)	235 (43.8%)	
>3	429 (25.5%)	69 (23.2%)	83 (20.4%)	118 (26.5%)	159 (29.7%)	
Parous	882 (52.3%)	160 (53.7%)	211 (51.8%)	232 (52.1%)	279 (52.1%)	0.96
Previous preterm delivery	292 (17.3%)	37 (12.4%)	57 (14.0%)	82 (18.4%)	116 (21.6%)	<0.01
Medicaid	751 (44.5%)	63 (21.1%)	142 (34.9%)	220 (49.4%)	326 (60.8%)	<0.01
Non-English speaker	88 (5.2%)	3 (1.0%)	18 (4.4%)	25 (5.6%)	42 (7.8%)	<0.01
Married	916 (54.3%)	206 (69.1%)	241 (59.2%)	219 (49.2%)	250 (46.6%) [n = 536]	<0.01
Substance use	28 (1.7%)	6 (2.0%)	6 (1.5%)	7 (1.6%)	9 (1.7%)	0.95

Q1 = Quartile 1, represents patients with lowest level of social vulnerability; Q2 = Quartile 2; Q3 = Quartile 3; Q4 = Quartile 4, represents patients with highest level of social vulnerability. * *p* value < 0.05 was deemed statistically significant.

**Table 2:** Betamethasone timing based on patient social vulnerability index quartile.

Betamethasone Timing	All (N = 1686) ^1^	Q1 (N = 298)	Q2 (N = 407)	Q3 (N = 445)	Q4 (N = 536)	*p* *
Optimal timing (betamethasone to delivery interval 2–7 days)	378 (22.4%)	48 (16.1%)	77 (18.9%)	115 (25.8%)	138 (25.7%)	
	Odds Ratios (95% CI)	Ref	1.22 (0.82, 1.81)	1.82 (1.25, 2.64)	1.81 (1.25, 2.60)	<0.01
	Adjusted Odds Ratios (95% CI)	Ref	1.08 (0.72, 1.63)	1.65 (1.11, 2.46)	1.49 (1.01, 2.22)	0.02
Betamethasone to delivery interval 6 h-7 days	606 (35.9%)	86 (28.9%)	128 (31.4%)	175 (39.3%)	217 (40.5%)	
	Odds Ratios (95% CI)	Ref	1.13 (0.82, 1.57)	1.60 (1.17, 2.19)	1.68 (1.24, 2.27)	<0.01
	Adjusted Odds Ratios (95% CI)	Ref	1.04 (0.74, 1.47)	1.56 (1.12, 2.19)	1.53 (1.09, 2.14)	<0.01
Betamethasone to delivery interval < 2 days	360 (21.4%)	59 (19.8%)	82 (20.2%)	90 (20.2%)	129 (24.1%)	
	Odds Ratios (95% CI)	Ref	1.15 (0.71, 1.89)	1.57 (0.98, 2.51)	1.31 (0.84, 2.06)	0.24
	Adjusted Odds Ratios (95% CI)	Ref	1.00 (0.60, 1.67)	1.29 (0.79, 2.13)	1.00 (0.61, 1.63)	0.51
Betamethasone to delivery interval < 6 h	132 (7.8%)	21 (7.1%)	31 (7.6%)	30 (6.7%)	50 (9.3%)	
	Odds Ratios (95% CI)	Ref	1.01 (0.54, 1.87)	1.42 (0.77, 2.63)	1.06 (0.60, 1.87)	0.55
	Adjusted Odds Ratios (95% CI)	Ref	0.87 (0.46, 1.65)	1.20 (0.63, 2.29)	0.82 (0.44, 1.52)	0.50
Betamethasone to delivery interval > 7 days	948 (56.2%)	191 (64.1%)	248 (60.9%)	240 (53.9%)	269 (50.2%)	
	Odds Ratios (95% CI)	Ref	1.15 (0.82, 1.60)	1.62 (1.18, 2.23)	1.79 (1.31, 2.45)	<0.01
	Adjusted Odds Ratios (95% CI)	Ref	1.07 (0.76, 1.51)	1.58 (1.12, 2.23)	1.64 (1.16, 2.32)	<0.01

^1^ N = 1686 for unadjusted odds ratios. N = 1667 for adjusted odds ratios due to missing data. Adjusted odds ratios adjusted for demographic variables in Table 1, with higher odds ratio indicating increased odds of optimal betamethasone timing; CI: confidence interval. Q1 = Quartile 1, represents patients with lowest level of social vulnerability; Q2 = Quartile 2; Q3 = Quartile 3; Q4 = Quartile 4, represents patients with highest level of social vulnerability. * *p* value < 0.05 was deemed statistically significant.

## Data Availability

The raw data supporting the conclusions of this article will be made available by the authors on request.

## Supplementary Materials

The following supporting information can be downloaded at: https://www.mdpi.com/article/10.3390/jcm13247798/s1, Table S1: Percent of patients with optimal timing of betamethasone by maternal demographic variables.
