# Supplementary material for: Effect of Social Vulnerability Index on Betamethasone Timing in Patients at Risk of Preterm Birth
Source: J Clin Med. 2024 Dec 20;13(24):7798. doi: 10.3390/jcm13247798 (PMC11727978; doi:10.3390/jcm13247798)
Supplement: Supplementary file 1 [file jcm-13-07798-s001.zip › jcm-3360596-supplementary.pdf]

Journal of Clinical Medicine

MDPI

Supplemental Table S1: Percent of Patients with Optimal Timing of Betamethasone by Maternal Demographic Variables

Category	Received Optimal Betamethasone	Odds Ratio (95% CI)	Adjusted Odds Ratio (95% CI)
**Maternal age**			
< 35	240 (22.6%) [n=1063]	Ref	Ref
35-39	95 (21.7%) [n=438]	0.95 (0.73, 1.24)	1.08 (0.81, 1.43)
40-44	39 (23.3%) [n=163]	1.04 (0.71, 1.54)	1.23 (0.81, 1.87)
>44	5 (22.7%) [n=22]	1.01 (0.37, 2.76)	1.12 (0.39, 3.18)
**BMI**			
<25	45 (21.3%) [n=211]	Ref	Ref
25-29	96 (17.9%) [n=536]	0.80 (0.54, 1.20)	0.78 (0.52, 1.17)
30-34	125 (24.7%) [n=506]	1.21 (0.82, 1.78)	1.12 (0.75, 1.66)
35-39	59 (23.6%) [n=250]	1.14 (0.73, 1.77)	1.01 (0.64, 1.59)
>=40	49 (29.7%) [n=165]	1.56 (0.98, 2.49)	1.40 (0.86, 2.27)
**Race/ethnicity**			
non-Hispanic White	90 (18.2%) [n=496]	Ref	Ref
non-Hispanic Black	120 (25.6%) [n=469]	1.55 (1.14, 2.11)	1.16 (0.82, 1.63)
Asian or Pacific Islander	57 (19.0%) [n=300]	1.06 (0.73, 1.53)	0.90 (0.61, 1.32)
Other/multiracial/ declined/unknown	111 (26.4%) [n=421]	1.62 (1.18, 2.21)	1.28 (0.91, 1.79)
**Gravidity**			
1	116 (24.7%) [n=470]	Ref	Ref
2	97 (20.2%) [n=481]	0.77 (0.57, 1.05)	0.86 (0.60, 1.23)
3	61 (20.0%) [n=305]	0.76 (0.54, 1.08)	0.95 (0.62, 1.44)

>3	104 (24.2%) [n=429]	0.98 (0.72, 1.32)	1.39 (0.91, 2.14)
**Parity**			
0	202 (25.1%) [n=804]	Ref	Ref
1	117 (21.2%) [n=553]	0.80 (0.62, 1.04)	0.80 (0.57, 1.11)
>1	59 (17.9%) [n=329]	0.65 (0.47, 0.90)	0.46 (0.30, 0.72)
**Previous preterm delivery**	62 (21.2%) [n=292]	0.92 (0.68, 1.25)	0.94 (0.67, 1.33)
**Medicaid**	187 (24.9%) [n=751]	1.29 (1.03, 1.62)	1.15 (0.88, 1.50)
**English proficiency**	354 (22.2%) [n=1598]	0.76 (0.47, 1.23)	0.81 (0.48, 1.38)
**Married**	183 (20.0%) [n=916]	0.74 (0.59, 0.93)	0.91 (0.70, 1.17)
**Substance use**	5 (17.9%) [n=28]	0.75 (0.28, 1.98)	0.72 (0.27, 1.96)
Adjusted odds ratio adjusted for SVI, maternal age, race/ethnicity, BMI, insurance status, marital status, primary patient language, gravidity/parity, history of preterm delivery, gestational age at timing of initial betamethasone, substance/alcohol use; CI=Confidence Interval			
